# Fe Core–Carbon Shell Nanoparticles as Advanced MRI Contrast Enhancer

**DOI:** 10.3390/jfb8040046

**Published:** 2017-10-09

**Authors:** Rakesh P. Chaudhary, Kim Kangasniemi, Masaya Takahashi, Samarendra K. Mohanty, Ali R. Koymen

**Affiliations:** 1 Department of Physics, University of Texas at Arlington; Arlington, TX 76019, USA; rakeshpratapbhai.chaudhary@mavs.uta.edu; 2 Advanced Imaging Research Center, Radiology, University of Texas Southwestern Medical Center, Dallas, TX 75390, USA; kim.h.kangasniemi@exxonmobil.com (K.K.); masaya.takahashi@utsouthwestern.edu (M.T.); 3 NanoScope Technologies LLC, Bedford, TX 76022, USA; smohanty@nanoscopetech.com

**Keywords:** MRI, contrast agent, superparamagnetic, core-shell nanoparticles

## Abstract

The aim of this study is to fabricate a hybrid composite of iron (Fe) core–carbon (C) shell nanoparticles with enhanced magnetic properties for contrast enhancement in magnetic resonance imaging (MRI). These new classes of magnetic core–shell nanoparticles are synthesized using a one-step top–down approach through the electric plasma discharge generated in the cavitation field in organic solvents by an ultrasonic horn. Transmission electron microscopy (TEM) observations revealed the core–shell nanoparticles with 10–85 nm in diameter with excellent dispersibility in water without any agglomeration. TEM showed the structural confirmation of Fe nanoparticles with body centered cubic (bcc) crystal structure. Magnetic multi-functional hybrid composites of Fe core–C shell nanoparticles were then evaluated as negative MRI contrast agents, displaying remarkably high transverse relaxivity (*r*_2_) of 70 mM^−1^·S^−1^ at 7 T. This simple one-step synthesis procedure is highly versatile and produces desired nanoparticles with high efficacy as MRI contrast agents and potential utility in other biomedical applications.

## 1. Introduction

Nanocrystals with enhanced magnetic properties have been actively pursued for potential biomedical applications such as drug delivery [1,2], hyperthermia therapy [3], magnetic resonance imaging (MRI) contrast enhancement [4,5,6,7,8,9,10,11,12], as well as nanotechnology applications such as catalysis and permanent magnets [13,14]. In order to use these nanoparticles for in vivo or in vitro biomedical applications such as MRI contrast enhancement, the combined properties of high magnetic saturation, stability, biocompatibility, and interactive functions at the surface are essentially required. Currently, iron oxide nanoparticles (IONPs) with various microstructure and magnetic properties have been extensively studied for use in MRI [15,16]. Preparation strategies of surface functionalized and core–shell IONPs are limited by the significant number of synthetic steps involved, leading to multi-step complexity [17,18].

In comparison to IONPs, iron (Fe) has superior magnetic properties, such as high saturation magnetization which is roughly double that of most of the iron oxides, and lower coercivity than IONPs. Hence, Fe nanocrystals will have a much stronger shortening effect on spin–spin transverse relaxation time (*T*_2_) than IONPs, suggesting that Fe nanoparticles may be a more effective contrast enhancer for MRI. Higher magnetization of Fe than IONPs will also ensure accurate dosage due to increased interactions with applied magnetic field [19]. However, it has yet to be explored owing to the problems of easy oxidation and potential toxicity. Although most studies to-date have focused on the development of polymer or silica protective coatings, recently carbon-protected magnetic nanoparticles are receiving more attention [18,20]. Carbon coating provides an effective oxidation barrier, prevents corrosion in magnetic core, and mitigates particle attraction, thereby avoiding agglomeration [21]. Fe core-coated C shell can thus endow these magnetic nanoparticles with biocompatibility, and higher chemical and thermal stability in many organic and inorganic media compared to bare Fe or IONPs [20,22]. 

In this paper, we present a one-step versatile approach to synthesize C shell-coated Fe core nanoparticles using plasma and their characterization. Biocompatible and stable multi-functional core–shell nanoparticles were explored by characterizing the magnetic properties of Fe core for contrast enhancement in MRI. Spin–spin transverse relaxation time (*T*_2_) of hydrogen proton in aqueous solution (water) of C coated Fe nanoparticles were evaluated as a contrast agent in MRI. The experimental observations suggest that the C coated Fe nanoparticles are a superior *T*_2_ contrast agent with an enhanced relaxivity of 70 mM^−1^·S^−1^.

## 2. Results and Discussion

**Figure 1:**
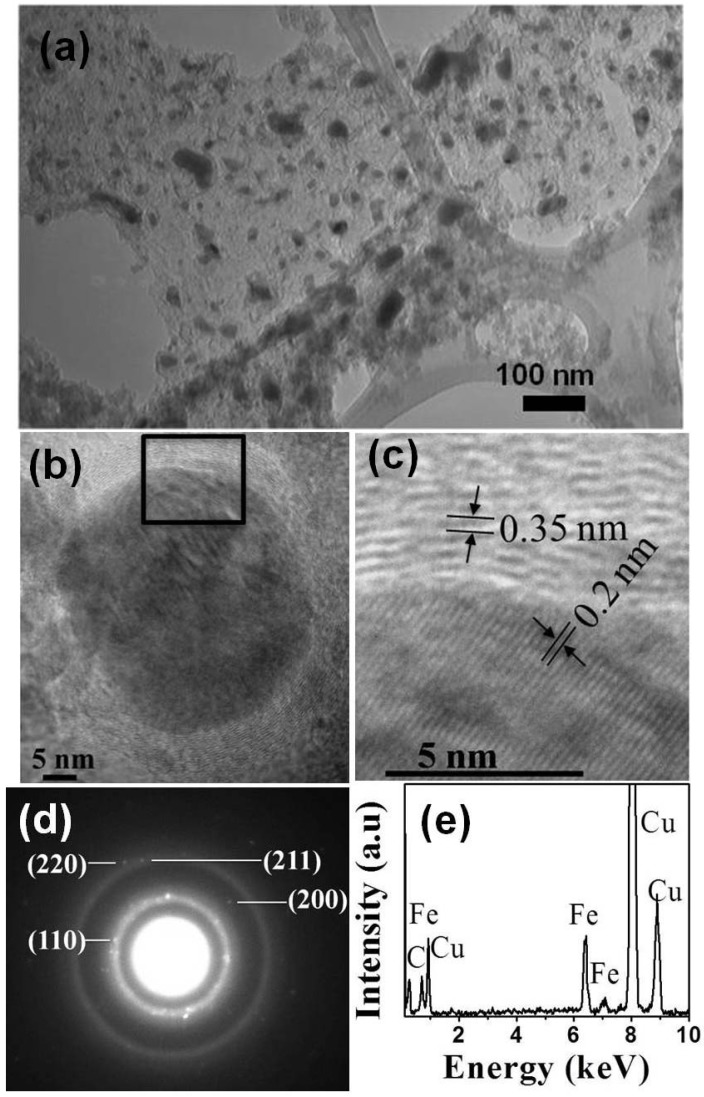
(**a**) TEM image of iron (Fe) core–carbon (C) shell nanoparticles; (**b**) HRTEM image of a single Fe-C nanoparticle; (**c**) Expanded view of the highlighted area in HRTEM image shown in (**b**); (**d**) Selected area electron diffraction (SAED) pattern of Fe-C nanoparticles; and (**e**) energy-dispersive X-ray spectroscopy (EDX) spectrum of the nanoparticles.

Nanoparticles size, morphology, chemical composition, and crystal structure were investigated using conventional TEM. Bright field TEM image (Figure 1a) shows polydispersed dark Fe nanoparticles with several layers of C shell. The smaller spherical nanoparticle diameters ranged from 10 to 44 nm. The elongated anisotropic nanoparticles were 25–34 nm in diameter and 50–85 nm in length. It is clearly visible that core–shell nanoparticles endow better stability and dispersibility, and hence prevent agglomeration. HRTEM (Figure 1b) shows one such particle with Fe core encapsulated by C shells. The shells are uniform in thickness and consist of 20–22 layers (7–8 nm thick). The spacing of the lattice fringes of C shell is about 0.35 nm (Figure 1c), which is close to that of the graphite (002) plane. The Fe cores have lattice fringe spacing of 0.203 nm (Figure 1c) related to the (110) plane of the bcc-Fe (PDF#06-0696). The number of covering layers depended on the particle size; small particles less than 20 nm in diameter were covered by three to four layers, whereas large particles had about 10–25 layers of C encaging them.

We identified a crystalline Fe core by selected area electron diffraction (SAED) analysis, shown in Figure 1d. The known characteristic lattice (d) spacings of Fe phase permit the unambiguous identification of the encapsulated material. The lattice spacings from the SAED pattern are 0.203 nm, 0.143 nm, 0.117 nm, and 0.101 nm, which correspond to the reported values of 0.2027 nm (110), 0.1433 nm (200), 0.117 nm (211), and 0.1013 nm (220) for Fe crystal structure (PDF#06-0696), respectively. Thus encaged crystals were clearly identified as the bcc phase of metallic Fe. Elemental compositions of the nanoparticles were analyzed using energy-dispersive X-ray spectroscopy (EDX). The EDX spectrum of nanoparticles exhibiting the characteristic peaks associated with Fe and C is shown in Figure 1e. No characteristic peaks of oxygen were evident. The presence of Cu is due to the TEM grid.

**Figure 2:**
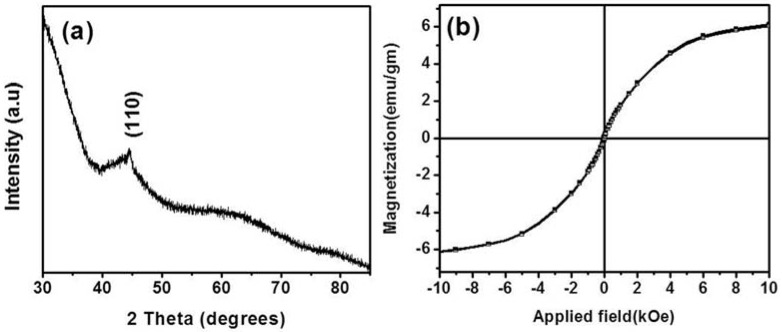
(**a**) XRD pattern of the nanoparticles; and (**b**) Magnetic hysteresis loop of the nanoparticles.

The XRD pattern of the nanoparticles is shown in Figure 2a. The poorly crystalline nature of the core–shell nanoparticles is demonstrated by the presence of only one diffraction peak. The peak at a 2θ value of 44.56° corresponds to the (110) lattice planes of bcc Fe. Figure 2b shows a magnetic hysteresis loop for the synthesized nanoparticles. Magnetic properties were measured at room temperature using a vibrating sample magnetometer (VSM) by applying an external magnetic field in the −8 kOe to +8 kOe range. The nanoparticles are superparamagnetic and the saturation magnetization (M_s_) for the nanoparticles was 9 emu/gm. The deviation of the magnetic properties of the nanoparticles is due to the proportion of nonmagnetic phase (carbon) and the poor crystalline nature of the Fe core of the nanoparticles.

**Figure 3:**
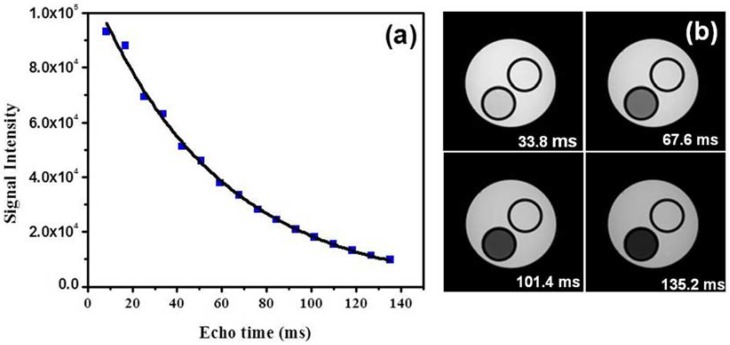
(**a**) Magnetic resonance imaging (MRI) signal intensity at different echo times for 0.326 mM concentration and (**b**) MR images recorded at different echo times 33.8, 67.6, 101.4, and 135.2 ms.

The transverse (*T*_2_) relaxation times of protons in water solutions of Fe-C nanoparticles were measured using a 7 T small animal MRI system. Phantoms of Fe-C nanoparticles of different concentrations were prepared from stock solution. To measure *T*_2_, a multi-echo spin echo sequence was used with repetition time (TR) of 4000 ms. The echo time (TE) was increased in 15 equal steps from 8.45 ms to 135.2 ms, as shown in Figure 3a. MRI images (Figure 3b) shown here were recorded at different echo times: 33.8, 67.6, 101.4, and 135.2 ms. A set of these *T*_2_-weighted multi-echo spin echo images (Figure 3b) showed the strong *T*_2_ contrast (dark contrast) from Fe-C nanoparticles in comparison to the H_2_O phantom. *T*_2_ of Fe-C nanoparticles were determined by fitting MRI signal intensities at different echo times (Figure 3a) using an exponential relation expressed as

(1) $$I=\;M_{0}\;exp^{-TE/T_{2}}$$

**Figure 4:**
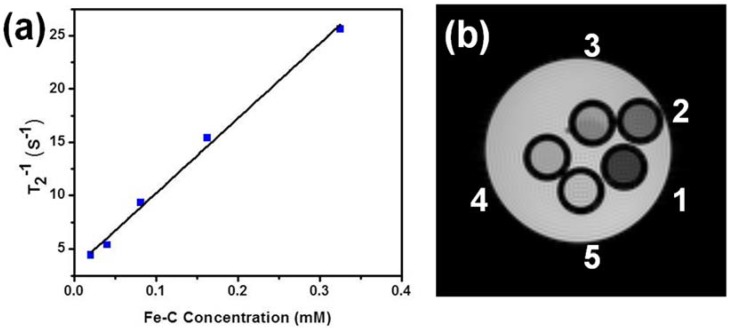
(**a**) *T*_2_^−1^ vs. Fe-C nanoparticle concentration; and (**b**) *T*_2_ weighted spin echo MR images for five different concentrations of the same samples: (1) 0.326 mM, (2) 0.163 mM, (3) 0.081 mm, (4) 0.040 mM, (5) 0.020 mM.

The efficiency of a negative contrast agent in changing the transverse relaxivity of nuclear spins in an aqueous solution of magnetic particles is expressed as

(2) $$\frac{1}{T_{2}}=\frac{1}{T_{2,O}}+r_{2}\left[Fe\right]$$

where *T*_2,0_ is the relaxation time of the medium in the absence of contrast agent, [Fe] is the concentration of iron, and *T*_2_ is the relaxation time of the medium with contrast agent. The transverse relaxivities (*r*_2_) were calculated from the slope of linear correlation between relaxation rate (1/*T*_2_) and concentration of Fe-C nanoparticles (NPs) (Figure 4a). The calculated *r*_2_ was 70 mM^−1^·S^−1^. Recent studies of C-coated IONPs with average diameter of 90 nm reported a relaxivity of 1.14 mM^−1^·S^−1^ [18]. The high relaxivity which is about 60 times greater in our results is attributed to the smaller average particle size of 22 nm and Ms of the Fe core. Shortening of the *T*_2_ relaxation time leads to spin dephasing, which generates dark contrast as seen in *T*_2_ weighted MRI images (Figure 3b). Figure 4b shows the imaging contrast of the samples containing different concentrations. There is a decrease in MRI signal in *T*_2_ weighted images (Figure 4b) with decrease in Fe concentration. Concentration-dependent negative enhancement in contrast is clearly visible. This study demonstrates that biocompatible and stable Fe core–C shell nanoparticles are possibly superior compared to currently used nanoparticles as contrast enhancers in MRI. We aim to localize these nanoparticles in targeted tissue regions by external DC magnetic fields, followed by MRI imaging and subsequent photothermal therapy [23]. Further, by varying the thickness of the carbon shell, we envision the use of the Fe core–Carbon shell nanoparticles for distance-dependent magnetic resonance tuning (MRET) [24] to image interactions between biological targets in vivo.

## 3. Materials and Methods

### 3.1. Synthesis of Core–Shell Nanoparticles

**Figure 5:**
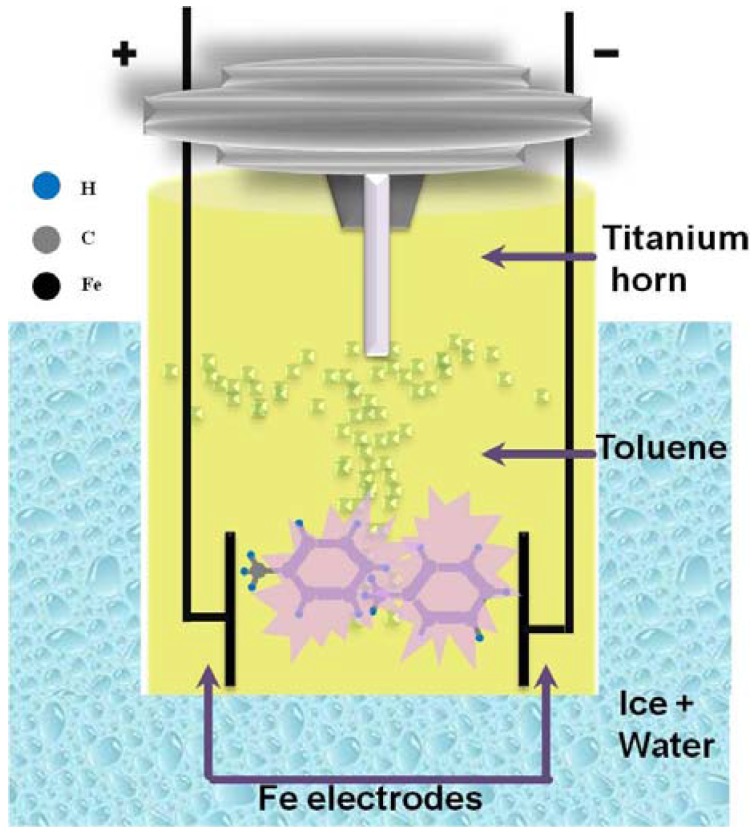
Schematic of the experimental set up that was used to synthesize core–shell nanoparticles.

Figure 5 shows a schematic diagram of the experimental apparatus used for Fe nanoparticle synthesis using a top-down approach. An ultrasonic processor (Sonics, VCX 750, Newtown, PA, USA) with a titanium horn (20 mm in diameter) was used to irradiate 275 mL of toluene (Sigma-Aldrich, anhydrous 99.8%, St. Louis, MO, USA) at 750 watts and 20 kHz. A glass vessel filled with ice water was underneath, and Argon gas flow was directed into the closed geometry containing toluene to maintain an inert atmosphere. As shown in Figure 5, two Fe electrodes (Alfa Aesar, 1 mm in diameter, 99.99%, Tewksbury, MA, USA) were inserted 0.5 mm apart from each other beneath the bottom of the ultrasonic horn. The distance between the electrodes and the bottom of the horn was kept constant at 10 mm. During the ultrasonic irradiation, the voltage between the electrodes was kept at 3 kV using a constant voltage power supply. Please add in the location of the manufacturer.

Ultrasound in toluene generates acoustic cavitations, which is the formation and implosive collapse of bubbles [25]. The collapse of cavitation bubbles in a strong electric field between Fe electrodes polarizes the π-electrons in toluene and thus creates plasma. Quasi-stable nanoparticles are formed when energetic plasma encounters electrodes either by nucleation in the vapor or molten Fe, and then after the rapid quenching, stable nanoparticles are formed [26,27]. The Plasma breaks toluene, and the C species are catalytically decomposed into C atoms. Since Fe is a known catalyst for graphitization, C atom forms a shell around Fe. 

### 3.2. Characterization of Core–Shell Nanoparticles

Nanoparticles were separated by centrifugation and washed in ethanol. Nanoparticles dispersed in ethanol using ultrasonication were dropped onto a carbon-coated copper grid and dried for the TEM investigation. The TEM (JEOL 1200 EX, operated at 120 kV, Peabody, MA, USA) observations allowed us to determine the overall morphology of the specimen and the crystal structure of the Fe nanoparticles. Fe nanoparticles were also investigated using high-resolution TEM (HRTEM, Hitachi H-9500, Dallas, TX, USA) operated at 300 kV. The crystal structure of nanoparticle powder was also determined using X-ray diffraction (XRD) with monochromatic Cu K_α_ radiation (Bruker D8 diffractometer, Billerica, MA, USA). The chemical composition of the nanoparticles was analyzed using energy-dispersive X-ray spectroscopy (EDX) linked with the TEM. The magnetic property measurements were carried out using a vibrating sample magnetometer (VSM) by applying a magnetic field of 8 kOe (Lakeshore, Model-7300, Westerville, OH, USA). Please add in the location of the manufacturer.

### 3.3. MRI Relaxometry

MRI was conducted using a 7-Tesla small animal MRI system (Varian Inc., Palo Alto, CA, USA) with a 40 mm radio frequency coil and a 400 mT/m gradient coil set. Fe-C nanoparticles 1 mg/mL concentration was diluted in deionized water in a tube (10 mL). Phantoms of different Fe-C nanoparticle concentrations were prepared from stock solution. A multi-echo spin echo sequence was performed for *T*_2_ measurements. Using a repetition time (TR) of 4000 ms, the echo time (TE) was increased in 15 equal steps from 8.45 ms to 135.2 ms.

## 4. Conclusions

We developed a new class of multifunctional Fe core–C shell nanoparticles of diameters 10–85 nm, and the average diameter of nanoparticles was 20 nm. Superparamagnetic core–shell nanoparticles in aqueous dispersion were explored for biological applications in MRI contrast enhancement. Fe core–C shell nanoparticles worked as a superior *T*_2_-weighted contrast agent for MRI with enhanced relaxivity of 70 mM^−1^·S^−1^. In comparison to currently used iron oxides and other metal elements, Fe core nanoparticles can achieve substantially higher relaxivity and hence enhanced MRI contrast. We believe C shell on Fe core improves the biocompatibility and stability of these nanoparticles compared to polymer or silica coatings. This study will open up new possibilities for C-coated magnetic nanoparticles in creating next-generation MRI contrast agents and MRET imaging probes. Since Fe core–C shell nanoparticles were found to be extremely non-reactive and highly absorbing in the near infrared regime, the development of C-based MRI contrast enhancement will allow its simultaneous use in other biomedical applications including near-infrared laser based photothermal therapy.
